# A zinc finger protein shapes the temperature adaptability of a cosmopolitan pest

**DOI:** 10.1098/rsob.240346

**Published:** 2025-04-09

**Authors:** Xin Miao, Fang Cao, Xiao-Fei Yu, Tian-Pu Li, Hai-Yin Su, Jiao Guo, Gui-Lei Hu, Bing-Wei Chen, Min-Sheng You, Yuan-Yuan Liu, Gao-Ke Lei, Shijun You

**Affiliations:** ^1^ State Key Laboratory of Ecological Pest Control for Fujian and Taiwan Crops, Fujian Agriculture and Forestry University, Fuzhou, Fujian, People’s Republic of China; ^2^ International Joint Research Laboratory of Ecological Pest Control, Fujian Agriculture and Forestry University, Fuzhou, Fujian, People’s Republic of China; ^3^ Ministerial and Provincial Joint Innovation Centre for Safety Production of Cross-Strait Crops, Fujian Agriculture and Forestry University, Fuzhou, Fujian, People’s Republic of China; ^4^ College of Life Sciences, Fujian Agriculture and Forestry University, Fuzhou, Fujian, People’s Republic of China; ^5^ Haixia Institute of Science and Technology, Fujian Agriculture and Forestry University, Fuzhou, Fujian, People’s Republic of China; ^6^ Institute of Plant Protection, Fujian Academy of Agricultural Sciences, Fuzhou, Fujian, People’s Republic of China

**Keywords:** *Plutella xylostella*, temperature adaptability, CRISPR/Cas9, age-stage two-sex life table

## Abstract

Global climate change is characterized by increased extreme temperatures affecting insects at all trophic levels. Zinc finger proteins (ZFPs) are key regulators of gene expression and cell differentiation in eukaryotes, essential for stress resistance in both animals and plants. Using CRISPR/Cas9 for gene deletion, this study predicted and examined the structure of ZFP320 in the diamondback moth (*Plutella xylostella*) and investigated its function in temperature stress response through a comprehensive age-stage, two-sex life table analysis. We found *ZFP320* encodes a 387 amino acid protein (43 kDa) with no transmembrane domains, featuring a ZnF-C2H2 domain. Quantitative fluorescence analysis showed that ZFP320 expression increased under high temperatures. ZFP320 knockout altered antioxidant gene expression, resulting in higher levels of superoxide dismutase and catalase in mutant strains compared with wild-type strain. Life table analysis revealed that the mutant strains had shorter fecundity and oviposition periods under both normal and high temperatures. Additionally, mutant strains exhibited lower parameters (*r*, *λ*, *R_0_*), as well as reduced survival rates and critical thermal maxima. Notably, *PxZFP320* plays a crucial role in temperature adaptation, paving the way for future investigations on the significance of ZFPs in *P. xylostella*’s temperature tolerance.

**Figure:**
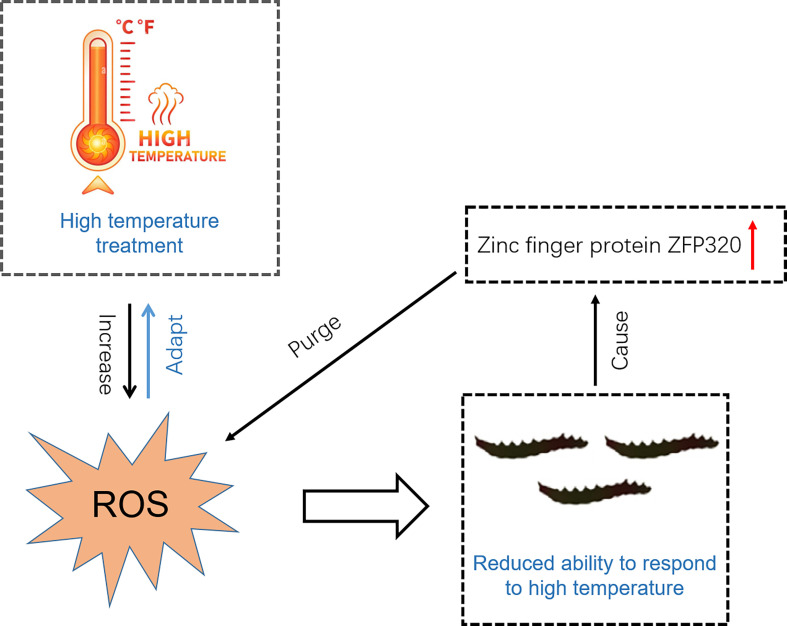

## 1. Introduction

Climate change has led to a significant surge in extreme temperature events and a persistent upward trend in global temperatures [1,2], posing a significant challenge to life forms, especially insects, whose survival depends heavily on ambient temperature to regulate biological processes [3,4]. These organisms, often lacking the physiological tolerance needed to withstand prolonged exposure to extremely high temperature [5,6], find themselves on the brink of extinction. Temperature, a vital abiotic factor, profoundly impacts insects’ physiological status and survival. Extreme temperatures can cause cellular disruption, cytoskeleton damage and protein synthesis interference [7,8]. Insects have evolved various mechanisms to adapt to this stressful environment, such as metabolic regulation and the production of protective proteins to mitigate or repair damage caused by high temperatures. One critical survival mechanism involving the induction of stress proteins is zinc finger proteins (ZFPs) [9]. The proteins produced by cells maintain intracellular protein homeostasis under stress conditions. ZFPs are critical stress-responsive proteins that help preserve cytoskeletal stability, prevent protein denaturation and facilitate the recovery of denatured protein structures and functions [10]. Previous studies have identified specific genes associated with insect resistance, such as ZFPs. These proteins are essential for the adaptability and resilience of insects in response to fluctuating climatic conditions, thereby aiding their survival in ever-changing ecosystems [11,12].

Zinc fingers are small structural motifs characterized by one or more coordinated zinc ions (Zn²^+^) and were first discovered in *Xenopus laevis* oocytes [13]. These motifs consist of eight distinct categories—Cys2His2 (C2H2), Treble clef, Zn2/Cys6, TAZ2 domain-like, Zinc ribbon, Zinc binding loops, Gag knuckle and Metallothionein—and are widespread across proteins. The C2H2 type, in particular, is commonly found in traditional ZFPs [14–16]. ZFPs exhibit a wide array of functions, including transcriptional regulation, apoptosis modulation, protein folding and assembly and lipid binding [17,18]. With the progress of research on their structure, newly discovered topological changes provide us with a deeper understanding of their biological importance. A noteworthy example is ZNF179, which is a neuroprotective agent that can reduce hydrogen peroxide-induced cell apoptosis and improve cell survival during oxidative stress. This discovery suggests that ZNF179 may be used for treatment to alleviate damage associated with reactive oxygen species (ROS) in nerve trauma [19,20]. The ZFP gene *AcZFP41* in *Apis cerana* (Hymenoptera: Apidae) is associated with oxidative stress response. In summary, ZFPs represent a diverse class of protein motifs and exhibit a wide range of biological functions. The extraordinary structural variations and functional complexity of these proteins continue to attract the interest of biologists. As we delve deeper into their interesting characteristics, we reveal innovative applications that may lead to transformative therapeutic strategies [21].

ZFPs play a crucial role in gene expression, cell differentiation and stress resistance in animals and plants [17]. These proteins regulate gene transcription through interactions with specific DNA sequences [18,22]. Zinc ions (Zn²^+^) play a crucial role in their function by activating sub-proteins within the zinc finger motif, enabling them to interact with enhancer sequences and regulate gene expression [23]. ZFPs are also involved in resisting various biotic and abiotic stress mechanisms [24]. In particular, they play a crucial role in the post-transcriptional regulation mechanism of heat stress [25].

The diamondback moth (DBM), *Plutella xylostella* (L.) (Lepidoptera: Plutellidae), is a notorious, destructive insect that mainly affects cruciferous crops and has a wide range of hosts. It poses a significant threat to global agricultural production [26,27]. It is concerning that due to its high genetic tolerance, *P. xylostella* has shown impressive potential in adapting to future climate conditions in different regions, making it a persistent and severe agricultural pest [28,29]. Insects like *P. xylostella* demonstrate remarkable resilience in the face of changing environmental conditions, particularly temperature fluctuations. They are able to evolve genetic variations that enhance their tolerance to extreme temperatures, enabling them to survive and thrive in previously unfavourable environments. This ability to adapt is pivotal in their success as a pest species [30]. Understanding the underlying genetic and physiological mechanisms that drive such adaptations holds the key to developing more effective pest control strategies. By identifying the genes and pathways involved in temperature tolerance, researchers can potentially target them with innovative pesticides or genetic engineering techniques, disrupting the moths’ survival under extreme conditions [31,32]. Moreover, studying insect adaptations to extreme temperatures offers insights into the broader impacts of climate change on ecosystems. As climate change introduces novel and challenging environmental conditions, understanding how insects, such as *P. xylostella*, adapt can enhance our knowledge of the resilience and vulnerability of various species and ecosystems. Such studies provide deeper insights into the intricate relationships between species and their environments and how these relationships are shaped by global changes [33]. In summary, understanding the mechanisms by which insects adapt to extreme temperatures is essential for informing the creation of effective pest management strategies. It also plays a vital role in mitigating the impacts of climate change on ecosystems. By gaining a deeper understanding of how insects adapt to changing environmental conditions, we can build more resilient agricultural systems and conserve the biodiversity of our planet.

This study mainly focuses on the transcription factor ZFP320, which is a member of the C2H2 ZFP family and plays a crucial role in transcriptional regulation. By using qRT-PCR, we observed an increase in the transcription level of *PxZFP320* in *P. xylostella* under high temperature stress (electronic supplementary material, figure S1). To characterize its protein structure and understand its physiological role in heat stress, we used CRISPR/Cas9 technology. This study aims to establish a theoretical framework to elucidate the mechanism of heat adaptation in the DBM.

**Figure 1.**
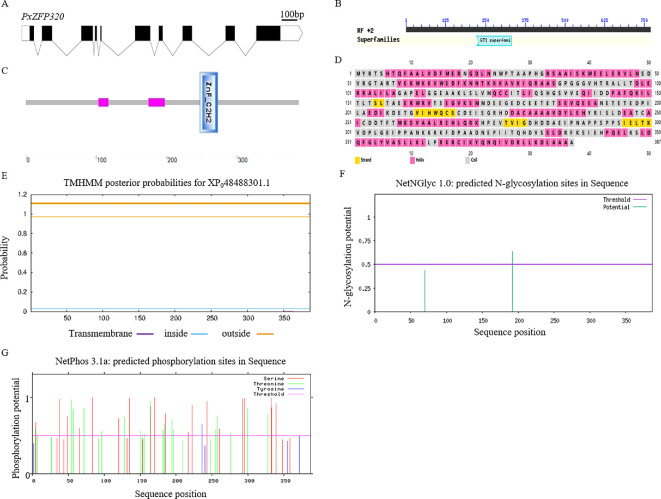
Gene and protein structure analyses of *PxZFP320* in *P. xylostella*. (A) Genomic structure of the *PxZFP320* gene. Black boxes indicate the exons; the spaces between two boxes are the introns, and the white boxes are the untranslated regions (UTRs). The figure is drawn to scale, and the scale bar is shown. (B) NCBI conserved domain database-based annotation of the deduced PxZFP320 protein sequence. (C) Protein domain prediction of PxZFP320 by SMART. The red indicates low complexity, the positions are 103−117 and 174−197, respectively. The blue is ZnF-C2H2, the position is 247−272. (D) Secondary structure of PxZFP320. Yellow represents strand; pink represents helix; grey represents coil. (E) The transmembrane domain of PxZFP320. (F) Predicted glycosylation of PxZFP320. (G) Predicted phosphorylation sites of *PxZFP320*.

## 2. Material and methods

### 2.1. Insect strains

In 2015, the Chinese Academy of Sciences’ Institute of Zoology kindly donated a wild strain of *P. xylostella*. It was kept under specific conditions in a climate-controlled facility: a 12 L : 12 D (photoperiod), a temperature of 26 ± 0.5°C and a humidity of 60 ± 5%. During adult mating, a 10% honey solution was provided as a source of nutrition.

### 2.2. Gene cloning

Amplification and sequencing of full-length cDNA were performed to obtain an accurate gene model. To extract total RNA, five larvae in their fourth instar (five duplicates) were combined and extracted using the Eastep® Super Total RNA Extraction Kit (Promega, USA). The agarose gel electrophoresis (1%) was used to evaluate integrity. The Reverse-Transcription System Kit (Promega, USA) was then used to create the cDNA template after a number of procedures.

The DBM genome sequencing database (http://59.79.254.1/dbm/index.php) provided the reference sequences for *PxZFP320*. SnapGene software was used to design primers (primers: *PxZFP320*-CDS-F; *PxZFP320*-CDS-R), as shown in electronic supplementary material, table S1. Using cDNA from the fourth-instar larvae as the template, the qualities of PCR products were confirmed through 1.5% agarose gel electrophoresis. The ligated construct was then transformed to DH5α cells, which were subsequently grown on LB solid culture medium supplemented with 100 μg ml^−1^ ampicillin and incubated at 37°C for 8 h. One colony was chosen and grown in 500 μl of LB liquid medium with 100 μg ml^−1^ ampicillin. The successful identification of a positive clone was achieved.

### 2.3. Analysis of sequences

The following tests were conducted to collect detailed information about gene function. The gene from the NCBI database was compared with the cDNA sequence of the *PxZFP320* that was produced by cloning. To acquire the genomic structure of *PxZFP320*, the Exon–Intron Graphic Maker (http://wormweb.org/exonintron) was used. NCBI was used to determine the conserved domains and protein-coding sequences of *PxZFP320*. We used the proteome prediction program Expasy to predict the relative molecular mass and isoelectric point of PxZFP320. Transmembrane regions and protein secondary structures were predicted using the TMHMM Server and PSIPRED, respectively. NetNGlyc 1.0 was used to predict the glycosylation location, and Net Phos 3.1 was used to predict the phosphorylation sites.

### 2.4. Patterns of *PxZFP320* expression in various tissues and developmental stages

The expression levels and distribution of *PxZFP320* in *P. xylostella* were examined in relation to tissue types and developmental phases. Total RNA was isolated from a variety of tissues (head, epidermis, midgut, Malpighian tubules, fat body and silk glands of fourth-instar larvae) and stages (eggs, first–fourth instars, pupae and adults of both female and male). For each sample, three technical and three biological duplicates were conducted. Electronic supplementary material, table S1 lists the qRT-PCR primers (primers: Q-*PxZFP320*-F; Q-*PxZFP320*-R), with the reference gene being RPL32 (Q-*PxRPL32*-F; Q-*PxRPL32*-R).

### 2.5. CRISPR/Cas9 genome editing for *PxZFP320* gene deletion

#### 2.5.1. Target screening for sgRNA

We used the CRISPR/Cas9 technique to create a mutant line of *P. xylostella* for the *PxZFP320* gene in order to confirm the functionality of *PxZFP320*. The Cas-OFFinder tool was used to assess the possible off-target effects of the sgRNA, and the target design adhered to the 5′-N20NGG-3′ guideline (with the PAM sequence highlighted). Within exon 3, a particular sgRNA recognition site was identified [34].

#### 2.5.2. Synthesis and purification of sgRNA

A single strand of nucleotides (primers: SgRNA-F; sgRNA-R) was used to create the sgRNA, as described in electronic supplementary material, table S1 [35]. The final product was removed and purified using gel extraction. We used the HiScribe T7 Quick High Yield RNA Synthesis Kit (New England Biolabs, USA) to generate the sgRNA. A phenol : chloroform extraction method was used to purify the sgRNA. The process outlined in §2.2 was followed in the approach for evaluating the product’s quality and concentration [36].

#### 2.5.3. Microinjection of the Cas9/sgRNA protein

A microinjection solution consisting of 300 ng μl^−1^ of sgRNA, 1 μl of Cas9 protein and 1 μl of 10× reaction buffer was prepared and adjusted to a final volume of 10 μl using nuclease-free water. After 30 min incubation at 37°C, the prepared solution was then injected into eggs, and the injection was completed within 15 min. All hatched or dead eggs were recorded, and the hatched larvae were kept under the same conditions as described in §2.1 [34].

### 2.6. Screening for mutations

The detection primers (*PxZFP320JC*-F; *PxZFP320JC*-R) (electronic supplementary material, table S1) were used to detect mutations caused by CRISPR/Cas9. Generation 0 (G0) was designated for the fertilized eggs. Following egg laying, genomic DNA was retrieved from the G0 adults, and the resulting larvae were defined as G1 progeny. These eggs were reared into adults, which were subsequently mated with a wild-type (non-injected) counterpart. To confirm the genotypes and identify appropriate lines for retention (i.e. individuals displaying double peaks in their sequence chromatograms at the positions corresponding to the sgRNA target sites), inbreeding was continued until homozygous mutations were obtained in cases where heterozygous individuals appeared [37].

### 2.7. Expression level of genes linked to antioxidants, including *PxProsβ2*, *PxPREP*, *Px15-PGDH* and *PxSOD*

The antioxidant-related genes were examined using the RNA extracted from wild strain and three mutant strains of *PxZFP320* (MU−5, MU+7 and MU−8). The primers used in the quantitative reverse transcription PCR (qRT-PCR) assays are detailed in electronic supplementary material, table S1 (primers: Q-*PxProsβ2*-F; Q-*PxProsβ2*-R; Q-*PxPREP*-F; Q-*PxPREP*-R; Q-*Px15-PGDH*-F; Q-*Px15-PGDH*-R; Q-*PxSO*-F; Q-*PxSO*-R). The experimental method for qRT-PCR was consistent with §2.4.

### 2.8. Superoxide dismutase and catalase

A total of 100 mg insects (female and male adults) from the wild and mutant strains were collected for examining enzyme activities using the SOD and CAT assay kit (Suzhou Keming Biotechnology Co., Ltd, China). The experiments included four biological replicates and three technical repetitions.

### 2.9. Age-stage two-sex life table study

We created life tables of both wild and mutant strains to examine the impact of *PxZFP320* gene knockout on *P. xylostella* acclimation at various temperature settings. One hundred and twenty freshly produced eggs (less than 30 min) from the wild or mutant strain were exposed to either the normal temperature (26°C) or the high temperature (32°C : 27°C = 12 h : 12 h). All other environmental factors were identical to those described in §2.1. Forty eggs (three replicates) from each strain were placed in a 30 mm Petri plate and provided with the same artificial diet as previously mentioned, with food being changed daily. The survival rate and the amount of time to pupation were noted for both strains. Pupae were moved into a 1.5 ml centrifuge tube that had been punctured. To facilitate mating and oviposition, the newly hatched female and male adults were placed in a 650 ml (120 × 100 × 78 mm) lunch box and provided a 10% honey solution. The number of eggs deposited by female adults was counted every day until their death. Both male and female longevities were noted [38].

The age-stage two-sex life table (TWOSEX-MSChart, Ver: 9/20/2024) was used to compute and evaluate the life parameters of *P. xylostella* [39–41]. While longevity refers solely to the adult stage for females or males, lifespan included the entire life cycle of the insect, encompassing all developmental stages (eggs, larvae, pupae and males and females). The population parameters, including age-specific fecundity (*m_x_*), age-specific survival rate (*l_x_*) and age-stage specific survival rate (*S_xj_*), were determined. Additionally, the following population parameters were computed: *r* (the intrinsic rate of increase), *λ* (the finite rate of increase), *R_0_* (the net reproductive value) and *T* (the mean generation time) using the equations [40]:


$$l_{x}=\sum_{j=1}^{\beta }s_{xj},$$

$$m_{x}=\frac{\sum_{j=1}^{\beta }s_{xj}f_{xj}}{\sum_{j=1}^{\beta }s_{xj}},$$

$$R_{0}=\sum_{x=0}^{\infty }l_{x}m_{x},$$

$$\sum_{x=0}^{\infty }e^{-r\left(x+1\right)}l_{x}m_{x}=1,$$

$$\lambda =e^{r},$$

$$T=\left(\ln R_{0}\right)/r.$$

The methods described by Chi & Liu [40] were used to calculate the stable age-stage distribution (SASD), stable age distribution (SAD) and stable stage distribution (SSD). The total pre-oviposition period refers to the duration from birth to the first oviposition, whereas the adult pre-oviposition period [42] specifically refers to the pre-oviposition interval in adult females. The software TWOSEX-MSChart [41] was used for life table analyses. A bootstrap method with 100 000 bootstrap samples was used to calculate the standard errors for population parameters, including fecundity, lifespan and developmental time. Variations among the different treatments were assessed using the paired bootstrap test [43].

### 2.10. Reaction to high temperature and critical thermal maximum

We conducted the following studies to investigate how the moth’s response to intense heat was affected by the knockout of the *PxZFP320* gene. A total of 3200 pupae from either the wild or mutant strains were each placed into a 1.5 ml centrifuge tube that had been perforated to allow the emergence of both males and females. Newly emerged adults were collected for testing. Four replicates of each treatment were conducted, with each replicate comprising either 20 males or 20 females. Each individual was exposed to 42°C for 30, 60, 90, 120 and 150 min. After exposure, the treated moths were placed in an artificial climate box at 26°C for 24 h, and their survival rates were recorded. Any movement of the moth’s limbs (mouthpart, legs and antennae) was considered life [44].

This experiment used an improved heating apparatus, based on the design developed by Calosi *et al*. [45], to measure the critical high-temperature (CT_max_) threshold of *P. xylostella* (electronic supplementary material, figure S2). The beaker dimensions were 113 × 154 mm, and the heating rate was adjustable within the range of 0.1–99°C min^−1^. The CT_max_ temperature for *P. xylostella* was defined as the temperature at which the moth entered a critical state during continuous heating. This critical state is characterized by an inability to crawl or fly after spasms. Most insects exhibit a sudden spasm upon reaching a certain temperature and then become motionless at the bottom of the container, with their abdomens facing upwards, while a few remain with their backs up with their antennae and limbs trembling before death within a few seconds. During the experiment, individual *P. xylostella* were transferred into 2 ml centrifuge tubes, which were numbered for accurate recording of each moth’s critical temperature. After sealing, the 2 ml centrifuge tubes were placed inside 50 ml centrifuge tubes, which contained a temperature data logger (Omega, HH509) to monitor the actual temperature changes within the 2 ml tubes. A foam board covered the entire heating apparatus to minimize heat loss and facilitate direct visual observation of the moths’ activity. The temperature in the beaker was gradually increased from 30°C to the predetermined temperature, and continuous visual monitoring of each moth’s behavioural changes occurred throughout the heating process. Since the heating rate influences insect heat tolerance, a heating rate of 0.25°C min^−1^ was selected based on preliminary trials.

**Figure 2.**
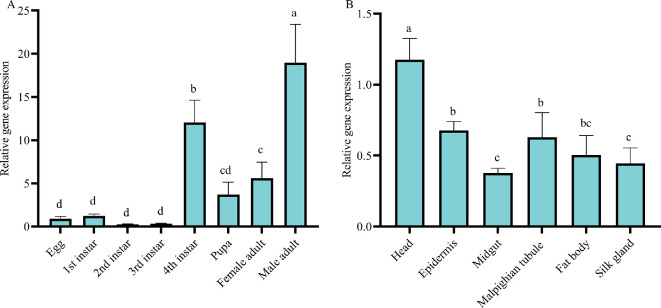
The expression patterns of *PxZFP320* in *P. xylostella*. (A) Expression of *PxZFP320* at different stages. (B) Expression of *PxZFP320* in different tissues of the fourth-instar larvae. Data are presented as mean ± standard error of the mean (s.e.m.), one-way ANOVA analysis was used for comparison (*p* < 0.05). Different letters indicate significant differences. (A) *n* = 3 biologically independent samples. (B) *n* = 4 biologically independent samples.

### 2.11. Statistical analyses

The relative expression of *PxZFP320* at various developmental stages and in different tissues was assessed using the 2^−ΔCt^ method [46]. Statistical analyses were performed using SPSS v. 26.0 (SPSS, USA). To examine the variations in the expression levels of antioxidant-related genes and the activities of CAT and SOD, one-way ANOVA was used between the wild-type and mutant strains, with least significant difference tests applied for multiple comparisons.

## 3. Results

### 3.1. *PxZFP320* identification and description

After analysing the *ZFP320* gene sequence of *P. xylostella*, it was found that its open reading frame is 1571 bp long, encoding a 387 amino acid protein of 43 kDa and isoelectric point of 5.08. It contains 20 common amino acids, without Sec, the highest Ala content (10.1%) and the lowest Val content (5.7%), with a fat solubility index of 84.01, an average hydrophilicity of −0.483 and an instability coefficient of 37.83. The *ZFP320* gene contains 10 exons and nine introns, showing a typical conserved domain of GT1 (figure 1A,B).

The SMART tool predicted that the ZFP320 contained ZnF-C2H2 domain (figure 1C). It was anticipated that the secondary structure of ZFP320 would primarily consist of random coils and an α-helix (figure 1D). The transmembrane structure prediction showed that the predicted values for each amino acid in *PxZFP320* were all greater than the threshold of 1, indicating that the protein is located entirely outside the membrane, lacks a transmembrane structure and is not a membrane protein (figure 1E).

The glycosylation site prediction results indicate that the N-glycosylation potential of the Asn-Xaa-Ser/Thr motif is above the threshold of 0.5, with a value of 0.637, suggesting the presence of N-glycosylation sites in *PxZFP320*. In contrast, the prediction of O-glycosylation sites showed that the potential values for all sites were below the threshold, indicating that no O-glycosylation sites are predicted (figure 1F).

The prediction of protein phosphorylation sites indicates that PxZFP320 has phosphorylation sites on three amino acid residues: Ser, Thr and Tyr. A total of 36 phosphorylation sites were identified, specifically Tyr^4^, Ser^5^, Thr^7^, Ser^38^, Ser^49^, Thr^54^, Thr^57^, Ser^66^, Thr^72^, Ser^84^, Thr^97^, Ser^120^, Thr^128^, Ser^135^, Thr^151^, Thr^156^, Thr^164^, Ser^165^, Ser^170^, Ser^182^, Thr^184^, Ser^185^, Thr^194^, Thr^196^, Tyr^217^, Tyr^222^, Ser^243^, Thr^255^, Thr^257^, Ser^260^, Thr^276^, Ser2^92^, Ser^295^, Thr^327^, Ser^333^ and Ser^339^ (figure 1G).

### 3.2. Patterns of *PxZFP320* expression in various tissues and developmental stages

We determined the spatiotemporal expression pattern of the *PxZFP320* gene in *P. xylostella* across various tissues and developmental stages. Our findings revealed that the expression level of *PxZFP320* was highest in male adults, followed by the fourth-instar larva (figure 2A). *PxZFP320* expression was highest in the head, with no significant differences observed among the epidemics, Malpighian tubule and fat body (figure 2B).

### 3.3. *PxZFP320* pure mutant strain

**Figure 3.**
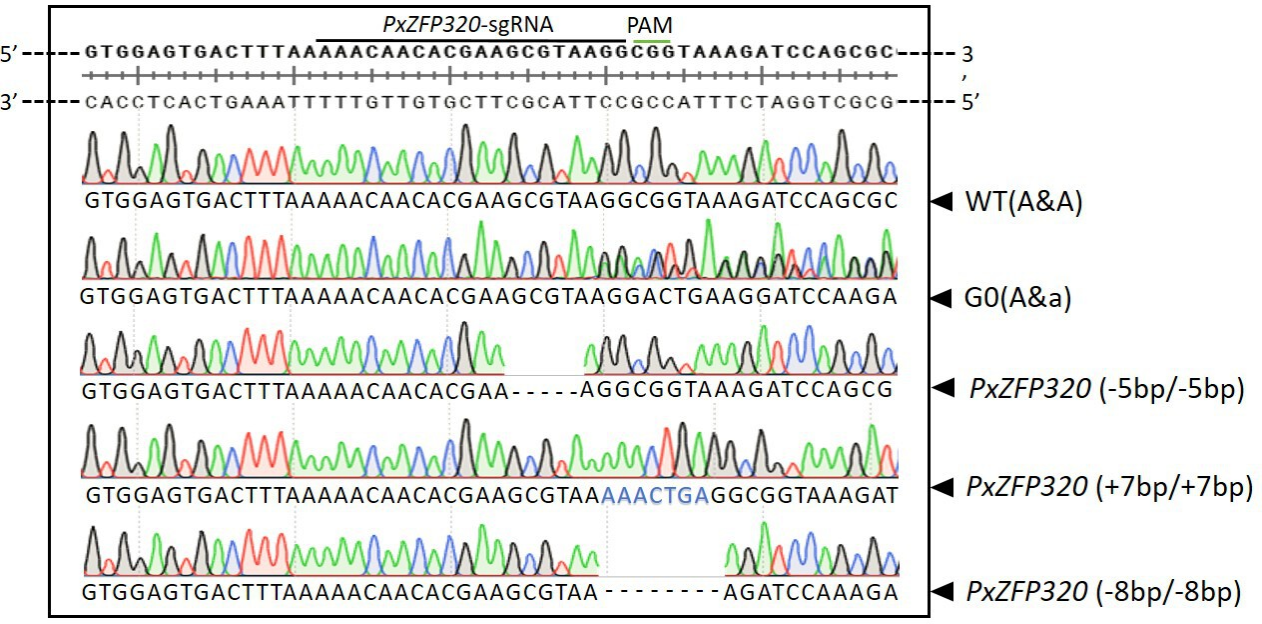
Targeted mutation of *P. xylostella PxZFP320* mediated by CRISPR/Cas9.

A combination of Cas9 protein (1 μl) and sgRNA (3000 ng μl^−1^) was administered to 400 newly hatched eggs of *P. xylostella*, resulting in a successful adult emergence rate of 41.0% (164/400), which were classified as the G0 generation. Following inbreeding of the G1 generation and subsequent egg laying, sequencing of the G1 generation identified three distinct mutant types: MU−5 bp, characterized by a five base pair deletion; MU+7 bp, featuring seven base pair insertions; and MU−8 bp, exhibiting eight base pair deletions. A sibling hybrid strain was created as a result of these mutation types being regularly observed in the G2 generation. In G3, sibling cross-pairs containing a single homozygote (aa × Aa) were formed, and in G4, stable homozygous mutants were verified. After the establishment of the mutant strains, they were propagated for over three generations to facilitate further experimental investigations (figure 3).

### 3.4. Antioxidant-related genes

**Figure 4.**
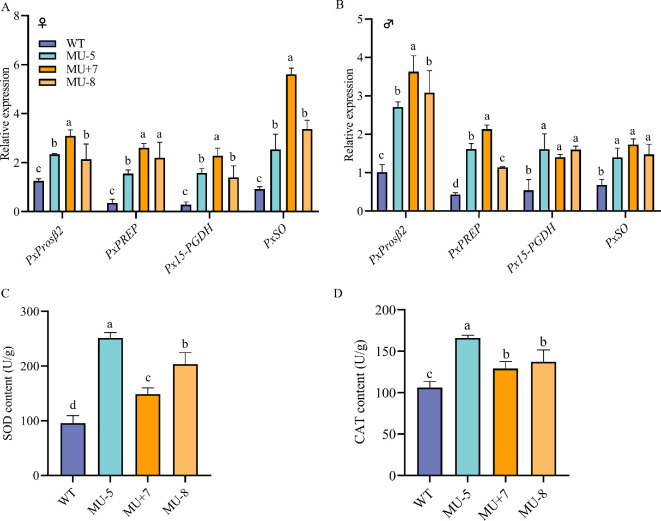
The expression level of antioxidant-related gene (*PxProsβ2*, *PxPREP*, *Px15-PGDH* and *PxSOD*) in female adults (A) and male adults (B), superoxide dismutase (C) and catalase activity (D) of wild and mutant strains of *P. xylostella*. Data are presented as mean ± s.e.m., and one-way ANOVA analysis was used for comparison (*p* < 0.05).

To investigate the function of *PxZFP320* in the defence against oxidative damage, we examined the mRNA expression profiles of a few antioxidant genes in three *PxZFP320* knockout mutant strains (MU−5, MU+7 and MU−8). As shown in figure 4, the expression levels of *PxProsβ2* (proteasome subunit beta type−7)*, PxPREP* (prolyl endopeptidase)*, Px15-PGDH* (15-hydroxyprostaglandin dehydrogenase [NAD (+)]) and *PxSO* (sulfite oxidase, mitochondrial) were all increased after the knockout of *PxZFP320* (figure 4A,B), indicating that the knockout of *PxZFP320* might lead to oxidative stress in *P. xylostella*.

### 3.5. Superoxide dismutase and catalase activity

Compared with the wild-type strain, we discovered that the mutant strains (MU−5, MU+7 and MU−8) had significantly increased superoxide levels (*t* = −16.062, *p* < 0.001; *t* = −5.169, *p* = 0.007; *t* = −7.4496, *p* = 0.003). The mutant strains’ (MU−5, MU+7 and MU−8) catalase activity was significantly higher than that of the wild-type strain (*t* = −12.702, *p* < 0.001; *t* = −3.499, *p* = 0.025; *t* = 3.389, *p* = 0.028) (figure 4C,D).

### 3.6. Age-stage two-sex life table

The duration of the egg and pupal stages was markedly shortened in mutant strains compared with that for the wild-type strain at both normal and high temperatures. Additionally, the fecundity and oviposition were also significantly shortened in mutant strains compared with that for the wild-type strain under both normal and high-temperature conditions (electronic supplementary material, tables S2, S3 and S6).

**Figure 5.**
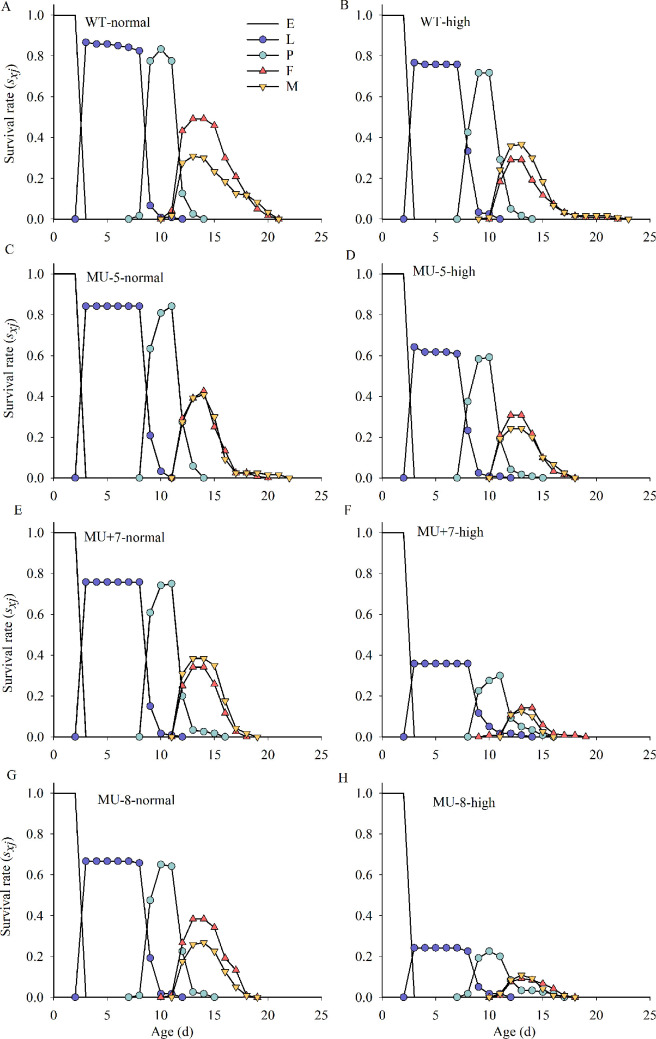
Age-stage survival rates (*S_xj_*) of wild and mutant strains of *P. xylostella* at different temperature environments. E, eggs; L, larva; P, pupa; F, female adult; M, male adult. Normal means at normal temperature (26°C); high means at high temperature (32°C/27°C).

Under normal temperature conditions (26°C), the survival rates for the juvenile developmental stages (larval and pupal phases) were as follows: the wild-type strain exhibited survival rates of 86%, while the mutant strains showed rates of 84, 76 and 67%. However, when exposed to high temperatures (32°C : 27°C = 12 h : 12 h), the wild-type strain’s survival rate decreased to 77%, compared with the mutant strains, which showed reduced survival rates of 64, 36 and 24% (figure 5).

**Figure 6.**
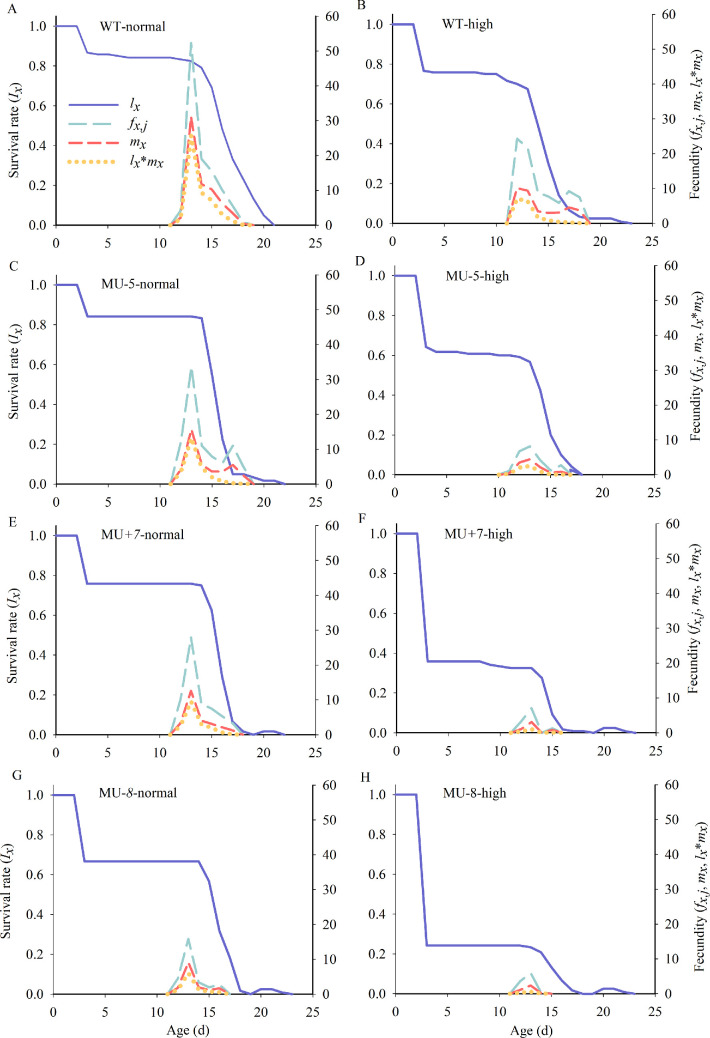
Age-stage survival rates (*l_x_*), female age-specific fecundity (*f_x,j_*) and age-specific fecundity total population of wild and mutant strains of *P. xylostella* at different temperature environments. Normal means at normal temperature (26°C); high means at high temperature (32°C/27°C).

The *l_x_* curve, which represents the variation in survival rates across different ages, indicated a significant reduction in survival during the pre-adult stage for the mutant strains. Additionally, the mutant strains had notably shorter lifespan under normal temperature conditions compared with the wild-type strain. The *f_x,j_* curve, illustrating daily egg production per female at age *x* and stage *j,* showed peak daily fecundities of 52.31 eggs for the wild-type strain, while the mutant strains produced 33.68, 27.90 and 15.83 eggs, respectively, at normal temperature. At high temperature, these figures dropped to 24.29 eggs for the wild-type strain and 8.18, 7.06 and 6.00 eggs for the mutant strains. The *m_x_* curve highlights the beginning and duration of the reproductive period. For both wild-type and mutant strains, the highest daily oviposition rates were observed on the 14th day at normal temperature and on the 13th and 14th days at elevated temperatures (figure 6).

Population parameters for the mutant strains demonstrated significantly lower intrinsic rate of increase (*r*), finite rate of increase (*λ*) and net reproductive rate (*R_0_*) when compared with the wild-type strain (electronic supplementary material, tables S4–S6).

**Figure 7.**
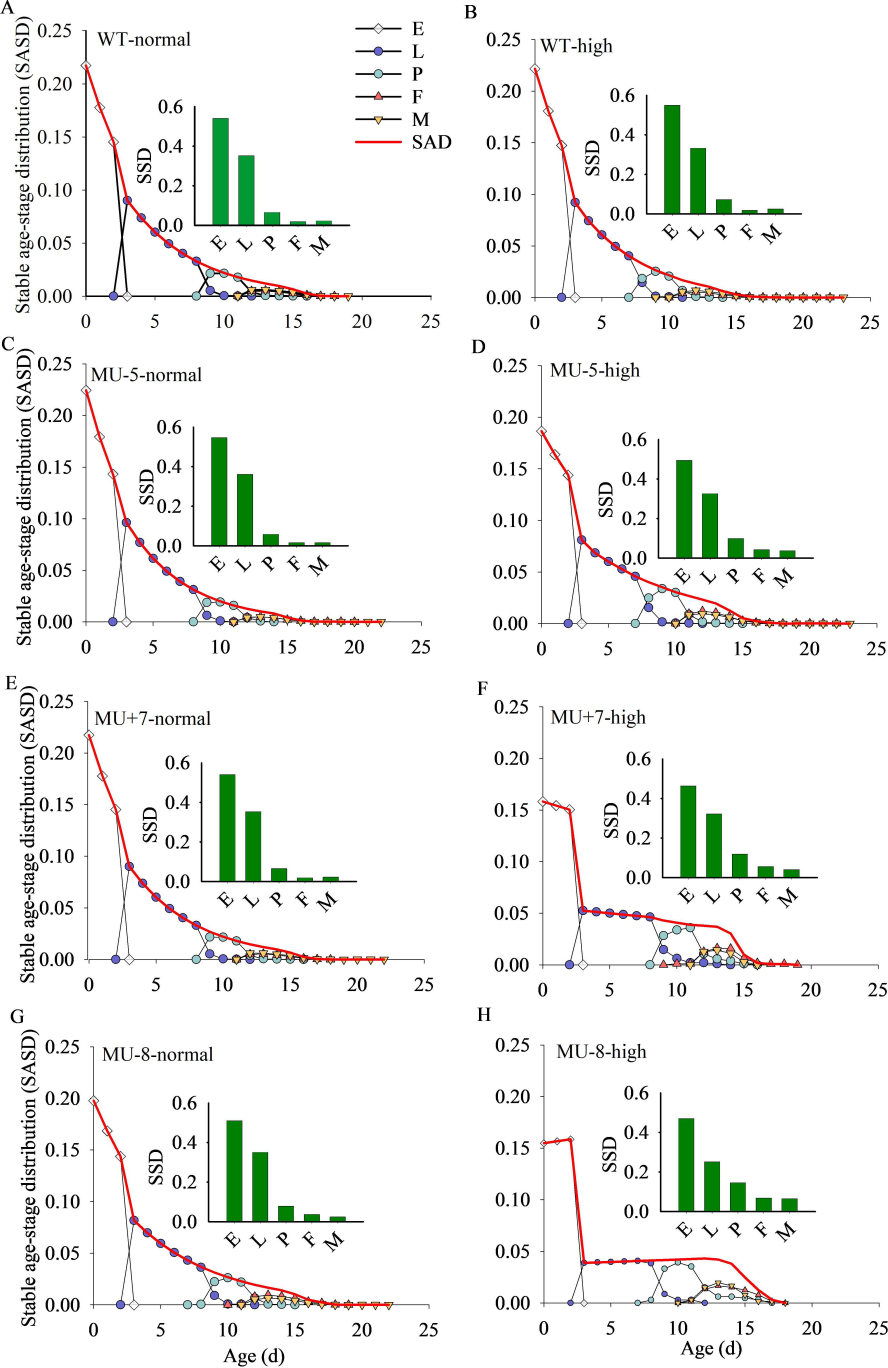
Stable age-stage distribution (SASD), stable age distribution (SAD) and stable stage distribution (SSD) of wild and mutant strains of *P. xylostella* at different temperature environments. Normal means at normal temperature (26°C); high means at high temperature (32°C/27°C).

The SASD, SAD and SSD are depicted in figure 7. At 26°C, the percentage of egg production was recorded at 54% for the wild-type strain, while the mutant strains showed egg production percentages of 36, 35 and 51%. Under high-temperature conditions (32°C : 27°C = 12 h : 12 h), the wild-type strain produced 55% of eggs, whereas the mutant strains produced 49, 46 and 47%.

**Figure 8.**
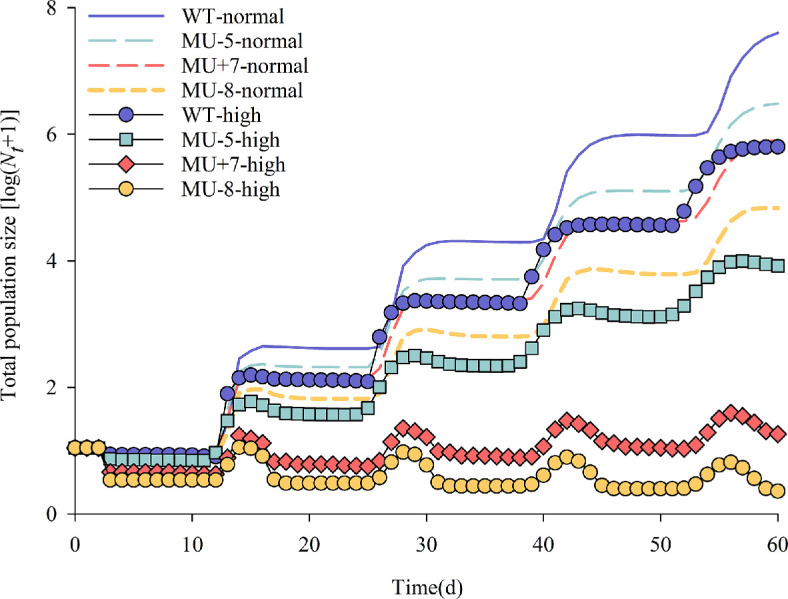
Stage structure curves of wild and mutant strains of *P. xylostella* at different temperature environments. Normal means at normal temperature (26°C); high means at high temperature (32°C/27°C).

For the population dynamics projection, both the wild-type strain and the mutant strains started with 120 effective eggs. After a 60 day projection period, four population generations were completed. Regardless of temperature conditions, the population prediction for the mutant strains was labelled as MU−5, MU+7 and MU−8 (figure 8).

### 3.7. Reaction to high temperature and critical thermal maximum

**Figure 9.**
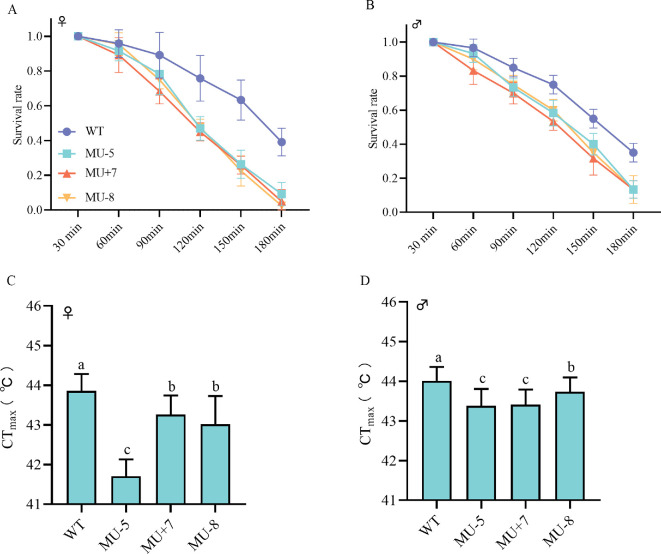
Response to high temperature of *P. xylostella* female adults (A) and female adults (B), critical thermal maximum of *P. xylostella* female adults (C) and female adults (D). Data are presented as mean ± s.e.m., and one-way ANOVA analysis was used for comparison (*p* < 0.05). Different letters indicate significant differences (*n* = 20) biologically independent samples.

As the duration of treatment at 42°C was prolonged, the survival rates of both male and female adults of the mutant strains decreased in comparison with those of the wild-type strain (figure 9A,B).

The critical thermal maximum (CT_max_) is a widely used and increasingly adopted metric for assessing an animal’s upper thermal tolerance threshold. The CT_max_ values for male and female adults of the mutant strains were found to be lower than those of the wild-type strain (figure 9C,D).

## 4. Discussion

Climate change has significantly exacerbated extreme high temperatures, exerting profound effects on insects at multiple biological levels [1]. This warming trend poses a considerable risk to insect populations, mainly by increasing their body temperatures [47]. To adapt to such stressful conditions, insects employ a range of survival strategies, with a particular focus on the synthesis of stress-resistant proteins. Among these proteins, heat shock proteins and ZFPs are particularly notable. Specifically, the upregulation of certain stress-resistant genes in response to thermal stress enhances insect tolerance [21]. The transcriptional regulatory roles of ZFPs are crucial in enabling insects to adapt to environmental stressors [48]. Thus, ZFPs play a vital role in helping insects navigate and survive various forms of stress, garnering significant interest within the research community.

ZFPs, characterized by their ZnF-C2H2 domains, play pivotal roles in transcription regulation within eukaryotes. These proteins interact specifically with DNA, RNA or protein sequences, regulating processes such as gene expression, cell differentiation, embryogenesis and stress responses. Their adaptability is crucial for maintaining cellular integrity across various ecosystems and in response to environmental stressors [49–53]. Sequence analysis of *PxZFP320* confirmed the presence of a ZnF-C2H2 domain, a hallmark of ZFPs. These proteins are essential for cellular integrity and survival, and their upregulation in response to stress enhances stress response mechanisms and adaptability in changing environments [54]. ZFPs are involved in vital processes such as growth, development, cell differentiation and stress responses in living organisms, emphasizing their importance in biology and biotechnology [55–57].

The amino acid profile of ZFP320 includes all 20 standard amino acids, with alanine (Ala) being the most abundant at 10.1%, while valine (Val) is the rarest at 5.7%. Ala, a neutral amino acid, serves as a crucial protein reserve and a primary humoral protein in fully developed insect larvae. The high Ala content may indicate a potential link to insect stress resistance [58]. Predictive analysis shows that ZFP320 lacks transmembrane domains, suggesting a nuclear-oriented function [59]. Notably, ZFP320 is expected to have 36 phosphorylation sites, primarily located on serine (Ser), threonine (Thr) and tyrosine (Tyr) residues. Post-translational modifications, such as glycosylation and phosphorylation, are essential for regulating protein structure, stability and functionality [60]. Studies have shown that N-glycosylation of TRPM8 ion channels can alter the temperature sensitivity of cold thermoreceptor neurons, while increased phosphorylation of AKT has been observed in response to elevated temperatures [61,62]. Therefore, N-glycosylation and phosphorylation are likely crucial for temperature adaptation. These findings suggest a complex interplay between amino acid composition, post-translational modifications and protein function in regulating biological processes, underscoring the importance of understanding the molecular mechanisms behind these interactions.

Our study revealed that *PxZFP320*, a ZFP, exhibits the highest expression levels in male adult *P. xylostella*, consistent with previous investigations in various Lepidoptera species [63]. This finding suggests that *PxZFP320* may play a critical role in physiological functions specific to male *P. xylostella*. Prior studies have established the significance of ZFPs in insect sex differentiation and reproductive development [64,65]. Through tissue-specific expression profiling, we discovered that *PxZFP320* is most abundantly expressed in the heads of fourth-instar larvae, which serve as critical nerve centres and endocrine organs in insects. Multiple studies have shown the involvement of ZFPs in neural development and hormone regulation, suggesting a potential role for *PxZFP320* in regulating growth and development in *P. xylostella* through neuroendocrine mechanisms [49,55]. Furthermore, although *PxZFP320* was also expressed in the epidermis, midgut and fat body, no significant differences in expression levels were observed. This widespread expression profile indicates that *PxZFP320* may modulate a range of physiological functions [56]. Previous studies have highlighted the role of ZFPs in various physiological processes in insects, including metabolism, detoxification and immune responses [50,51,57]. For instance, research by Li *et al*. [52] demonstrated that ZFPs interact with specific genes to promote melanized microsclerotia formation. Overall, our findings highlight the potential importance of *PxZFP320* in regulating physiological functions in *P. xylostella* and contribute to the growing body of knowledge on the role of ZFPs in insect biology.

Various animal species have been documented to undergo changes in their antioxidant and metabolic systems when exposed to thermal stress. To investigate the role of *PxZFP320* in responding to high temperatures, we created three mutant lines of *PxZFP320* using CRISPR/Cas9 technology. In our study, we subjected DBMs to elevated temperatures, which triggered a selective activation of *PxZFP320* expression (electronic supplementary material, figure S1). This discovery indicated that high temperatures induced intracellular oxidative stress through distinct pathways. Upon knocking out *PxZFP320*, we observed a significant increase in the expression of specific antioxidant genes, such as *PxProsβ2*, *PxPREP*, *Px15-PGDH* and *PxSO*, suggesting that these genes may respond differently to oxidative stressors and could be influenced by *PxZFP320*. Previous research has highlighted the pivotal role of ZFPs in regulating antioxidant-related gene expression. For example, enhanced tolerance to oxidative stress in *Arabidopsis* was observed by overexpressing the zinc finger protein ZAT12 [53], and in rice, a C2H2-type zinc finger transcription factor called ZFP182 [54] regulated antioxidant enzyme genes, thereby improving the antioxidant capacity of rice. Our study further revealed that the knockout of *PxZFP320* led to a marked increase in the activities of key antioxidant enzymes, CAT and SOD. SOD converts superoxide anions into hydrogen peroxide, while CAT further breaks down hydrogen peroxide into water and oxygen, offering protection against oxidative damage [66]. The increased activities of these enzymes following *PxZFP320* knockout suggest the induction of oxidative stress. Furthermore, reduced levels of ZFP37 in *A. cerana* resulted in increased SOD activity, underscoring the role of ZFP37 in maintaining redox balance [67]. Overall, our findings suggest that the knockout of *PxZFP320* triggers a substantial rise in ROS, leading to oxidative stress. In summary, our study positions *PxZFP320* as a critical player in response to oxidative stress.

Previous studies have demonstrated the significant impact of ZFPs on growth and development in various organisms. For example, Nazario-Yepiz & Riesgo-Escovar [68] showed that Piragua, a ZFP, is crucial for developmental processes in *Drosophila*. Similarly, Zhou *et al*. [69] found that the absence of the ZFP Rotund hinders thoracic leg development in *Bombyx mori* (lepidoptera: Bombycidae). Building upon these findings, our research focused on the knockout of *PxZFP320* and its effects on the development, reproduction and temperature adaptability of *P. xylostella*. Our observations revealed that the mutant strains displayed significantly shorter fecundity and oviposition periods compared with the wild-type strain, under both normal and high temperature conditions. Additionally, important population parameters such as *r*, *λ* and *R*_0_ were notably lower in the mutant strains compared with the wild-type strain. Our study suggests that the zinc finger protein ZFP320 in *P. xylostella* plays a critical role in influencing developmental duration and female fecundity. This highlights the significant contribution of genetic factors in the growth and development processes of the species. Furthermore, previous research identified the transcription regulator OVO as a key player in oocyte development [55]. In evaluating *PxZFP320*’s role in temperature adaptation, we conducted experiments exposing adult insects to extreme temperatures for varying durations. Our results indicated that the *PxZFP320* mutant strains exhibited reduced tolerance to high temperatures. This supports the established notion that ZFPs are crucial for temperature adaptation in insects [70]. Overall, our research reaffirms the vital role of transcription factors, such as ZFPs, in mediating temperature adaptability and influencing key aspects of growth and development in *P. xylostella.*

In conclusion, our integrated findings from gene expression quantification, CRISPR/Cas9 technology, enzymatic assays and two-sex life table studies demonstrate the potential function of *PxZFP320* in antioxidant defence mechanisms, linking it to developmental processes. High temperatures could lead to increased ROS, resulting in mortality in *P. xylostella*. Simultaneously, *P. xylostella* upregulated *PxZFP320* expression to mitigate oxidative damage induced by elevated temperatures. Therefore, we hypothesize that *PxZFP320* played a significant role in detoxifying ROS, facilitating *P. xylostella*’s adaptation to high-temperature environments. Our findings provide a valuable foundation for further investigations into antioxidant activity and thermal adaptability in this context.

## Data Availability

ZFP320 gene sequence: GenBank accession number XM_048632344. ZFP320 protein sequence: GenBank accession number XP_048488301. Structural features data: detailed analysis on the structural features of the ZFP320 protein, including primary structure, secondary structure predictions, hydrophilicity values and instability coefficients, is included as electronic supplementary material. Post-translational modification data: information on predicted glycosylation and phosphorylation sites for ZFP320 can be accessed through the electronic supplementary files [71].
